# Rise of the Med‐Ed‐ists: Achieving a critical mass of non‐practicing clinicians within medical education

**DOI:** 10.1111/medu.14940

**Published:** 2022-10-04

**Authors:** Helen Church, Megan Elizabeth Lincoln Brown

**Affiliations:** ^1^ Faculty of Medicine and Health Sciences University of Nottingham Nottingham UK; ^2^ Medical Education Innovation and Research Centre Imperial College London London UK; ^3^ University of Buckingham Buckingham UK

## Abstract

In the latest "Connections" article, Church and Brown use the concept of 'critical mass' to explain how stigma, lack of career pathways and prioritization of clinical seniority may discourage non‐practicing clinicians from becoming educationalists.

## 1 INTRODUCTION

‘If you're not willing to practice, there's no career for you in medical education’—these words were spoken to one of the authors of this article by a well‐respected physician during their medical education PhD. As we establish our own medical education careers, we, the authors, are passionate about proving this naysayer wrong, but the perception that non‐practicing, or ex‐, clinicians have no place in medical education is pernicious and pervasive.

Each year, 4% of UK doctors (around 5000 individuals) leave clinical medicine.^1^ Their plans and destinations are not systematically reported, though reasons including lifestyle choice, finding a passion elsewhere, training abroad and burnout are all cited.^2^ Medical education is something many ex‐clinicians have experience of and could represent a logical and fulfilling career path for many who choose to leave medicine. Indeed, Church and Agius^3^ report that many doctors taking career breaks after their first 2 years of training in the UK enter short‐term non‐clinical medical education roles, following which, the vast majority of doctors will then apply to higher clinical training programmes.

This early career decision demonstrates an appetite for medical education careers amongst trained doctors, yet the experiences of those who choose to exit clinical work completely for medical education are largely unknown. From our observations, only a minority of non‐practicing clinicians occupy visible medical education positions in long‐term, substantial medical education roles, suggesting that there lies a point somewhere between interest in medical education as a non‐clinical career and successfully transitioning to such a role that those with interest are dissuaded or prevented from progressing. Our experiences suggest that stigmatising perceptions regarding those who leave clinical medicine might be to blame, though our stories exist in a chasm within medical education literature. This connections article aims to examine recent papers within Medical Education to identify possible reasons why stigma regarding this career choice exists and ways in which we might begin to support those who choose this less well‐trodden path.

## 2 ARTICLE SUMMARY

### 2.1 Making the leap to medical education: A qualitative study of medical educators' experiences

Browne et al.^4^ explain that medical educators occupy a liminal space between their primary professional identity (as clinician or researcher), and medical educationalist, whereby the latter often holds less ‘social capital’. The lack of formal support for educators' professional identity transition is compensated by having a firm footing in any of the three facets of researcher, educator and practicing clinician, which may deter feelings of imposter syndrome and poor ‘credibility’. These issues are likely to dissuade those considering making the transition (leap) to becoming a medical educator.

### 2.2 Looking into the labyrinth of gender inequality: Women physicians in academic medicine

Han et al.^5^ describe the Catch‐22 of how academic women physicians are devalued based on their potential to have a family in both their clinical career (which discriminates against them to avoid workforce planning difficulties), and academic career (in which schools favour their more ‘retainable’ male counterparts as faculty). Leaving either career carries negative stigma and harms one's credibility, particularly as an early‐years doctor. The advice to overcome these hurdles includes ‘not requesting a privilege as a woman but working harder than male physicians’.

### 2.3 Approaching culture in medical education: Three perspectives

Watling et al.^6^ define three perspectives on medical educational culture. The organisational perspective unites the professionals working with it in pursuit of a common goal that is founded upon shared values and beliefs. The identity perspective highlights the need for promoting individuality and recognising that identity is multiple and fluid. Finally, the practical perspective focusses on ignoring material elements of medical education (people and things) and focusses on activity.

### 2.4 Job crafters

This commentary offers a fresh perspective on how individuals experience and exercise agency within medical practise. The authors^7^ suggest individuals exercise agency by engaging in ‘job crafting’, where they reflect and assign meaning to their work (cognitive crafting), make changes to the work they perform (task crafting), and prioritise certain relationships over others (relationship crafting). Trainees undertake job crafting when they sense misalignment between their identity and their experiences at work.

## 3 CONNECTIONS

To illustrate how these papers conceptualise non‐practicing clinicians and their role within medical education, we draw an analogy with the term ‘critical mass’. Within nuclear physics, a critical mass is the smallest amount of fissile material needed to sustain a nuclear chain reaction. The central tenet of this definition—the smallest amount necessary for a sustained reaction—has been adopted within popular culture to refer to any context in which things change after a certain number of people come together. However, within nuclear physics, material's surroundings (e.g., the temperature and pressure of an environment) are also critical to the chain reaction. In the medical education context, we argue that simply attempting to recruit those who leave healthcare professions is futile if the environment or ‘culture’ of medical education is not optimised for those individuals to succeed.

Our four papers all concern the nature of, or individuals' interactions with, the *culture* of medical education, which is largely unexplored in relation to the experience of non‐practicing clinicians. When considering the theory of culture, an organisational^6^ perspective describes the way in which working towards a common goal unites members of a group or field. Viewing our papers using this theoretical lens offers insights into three barriers ingrained in the current culture of medical education which lead to failure to support transition into, and progression within, medical education. By challenging these barriers and redefining our common goal, we aim to encourage a more sustainable and diverse community of educationalists.

The first of these barriers concerns the stigma of leaving clinical practice and making the leap into medical education. This is a particularly prominent barrier for women who, within the patriarchal labyrinth of career progression, struggle to find a firm footing within academia whilst maintaining clinical practice, but feel unable to step away from practice, as doing so can negatively impact one's credibility as an educator.^8^ Given this tension, women can become trapped in a liminal zone, oscillating between the identities of medical educationalist and clinician, identifying themselves as neither one nor the other, and sensing that they are not accepted wholly by clinicians or by non‐clinical educators. The fact that these phenomena are more pronounced for early‐career women,^9^ may explain why disparity exists between those interested in medical education immediately after leaving medical school and those securing formal roles later in their careers.

A lack of a clearly defined career pathway for non‐practicing clinicians within medical education is the second barrier to forming a critical mass. Medical education careers lack the traditional organisational structure of a training pathway—they are often serendipitous. This makes accessing the career and developing one's professional identity challenging.^9^ As Bochatay et al.^7^ explain, empowering clinical trainees to engage in job crafting increases their autonomy within identity development. At the most basic level, aspiring medical educationalists should be offered opportunities to engage in ‘task crafting’, for example, through welcoming their active involvement in teaching and research, or supporting them to obtain formal medical education qualifications that might grant them cultural capital in the field.^10^ ‘Cognitive job crafting’ is most difficult to support, as it involves individuals reframing their work to become meaningful. This can be challenging, given the stigma associated with leaving clinical practice, the value (or cultural capital) assigned to current practice and lack of career pathway. Hu et al.^10^ assert that, although the non‐clinical educators in their study were ‘unable to draw upon cultural capital accrued from clinical work’, they ‘built social capital through essential service relationships, capitalising on times when education takes precedence, such as curriculum renewal and accreditation’ to strengthen their position. In essence, finding an area of expertise within medical education may enable non‐practicing clinicians to claim back some of the capital lost upon leaving practice and promote cognitive job crafting. It is essential that mentors and leaders encourage non‐practicing clinicians to hone an area of expertise.

The third issue identified from our papers is the perceived superiority of clinical experience. Both Browne et al.^4^ and Hu et al.^10^ comment that clinical practice acts as cultural capital within medical education, where practicing clinicians are often appointed to positions of seniority without doctorates or educational expertise, over‐and‐above scholars with more field‐specific experience. Hu et al.^10^ argue that postgraduate degrees (e.g., PhDs) may act as another form of cultural capital, yet this runs contra to the experience of the authors of this article with lived experience in this area. Most individuals within medical education have a primary professional background, which affords them membership into multiple associations or communities which bind them through practice, be that clinical, research, educational or their primary academic training. For the few ex‐practitioners, their lack of current clinical practice invalidates their membership to medical organisations and so also to their identity claim to know anything about the way in which those within medical organisations should be educated. There is a pervasive lack of understanding regarding the valuable insight non‐practicing clinicians' past clinical experience, and subsequent non‐clinical immersion in educational practice and theory, can offer medical education.^6^

There are several steps we can take to challenge the toxic elements of medical education's organisational culture that prevent non‐practicing clinicians from flourishing. It is essential that we advocate for acceptance, and celebration, of diverse career backgrounds. This includes seeking and welcoming practicing, non‐practicing and non‐medically educated professionals. Leaders must prioritise the development of a diverse educational workforce and those responsible for recruitment should consider opening educational, research and leadership positions to all. ‘Relationship crafting’ can be enhanced through career mentorship initiatives. Funding should be made more widely available for non‐practicing clinicians to pursue formal qualifications. Finally, we suggest that positioning those working within medical education as ‘medical educationalists’ with unique expertise regardless of their background, and increasing the use of this label and status, might contribute to the development of a group identity that reduces liminality and stigma.

## 4 CONCLUSION

To reach a critical mass of non‐practicing clinicians within medical education, it is not enough to simply advertise medical education career paths to this group; we must work collectively to create a new, dynamic organisational culture that focusses on what an organisation *wants to achieve*, allows the value of non‐practicing medical educators to be realised and empowers those that transition into the field to craft meaningful professional identities that embrace their unique perspective. We hope this article draws attention to the experiences of this group within medical education, and that others in the community join us in exploring these experiences from a variety of perspectives. In doing so as a collective, we can reposition non‐practicing clinicians within medical education, opening the door of our field more widely to those interested and respecting and valuing those already admitted.

## CONFLICT OF INTEREST

The authors declare no conflict of interest.

## ETHICS STATEMENT

Ethics approval was not required for this study.

## AUTHOR CONTRIBUTIONS

HRC had the initial idea for the article, before both HRC and MELB researched potential papers for the article. Both authors wrote substantial parts of the manuscript, edited and revised the work and agreed on final version prior to submission. Both authors are accountable for the integrity and accuracy of the article.
